# Predictive factors and clinical outcomes following contemporary percutaneous coronary interventions for chronic total occlusions

**DOI:** 10.3389/fcvm.2026.1762032

**Published:** 2026-03-19

**Authors:** Li Jin, Chen Youhu, Wang Huan, Chen Genrui, Lei Xiaolin, Cai Yue, Li Chengxiang, Gao Haokao

**Affiliations:** Department of Cardiology, The First Affiliated Hospital of Air Force Military Medical University, Xi’an, China

**Keywords:** chronic total occlusion (CTO), coronary angiography (CAG), major adverse cardiovascular events (MACE), percutaneous coronary intervention (PCI), target vessel revascularization (TVR)

## Abstract

**Background:**

We aimed to explore the predictive factors and one-year clinical outcomes following contemporary percutaneous coronary intervention for chronic total occlusion lesions (CTO-PCI).

**Methods:**

Consecutive patients from January 2018 to December 2020 were enrolled. The primary endpoint was major adverse cardiovascular events (MACE), which included all-cause death and myocardial infarction, while target vessel revascularization (TVR) was defined as the secondary endpoint, all of which were measured at 12 months.

**Results:**

891 consecutive patients underwent 1,010 CTO PCIs. Of these 871 patients meeting the inclusion criteria accepted the index CTO PCI and 96.8% of these cases were available with one-year follow-up data (*n* = 843). The incidence of MACE was observed in 29 patients (3.4%), with all-cause death occurring in 22 cases (2.6%) and myocardial infarction in 7 cases (0.8%). TVR occurred in 39 patients (4.6%). The multivariate analysis for MACE indicated that LVEF < 40% (*P* = 0.001) and residual SYNTAX score ≥12 (*P* = 0.020) was identified as independent risk factors. Parallelly, proximal ambiguity cap (*P* = 0.008) and increased guide-wire manipulation time (*P* < 0.001) were independent risk factors for TVR.

**Conclusion:**

Patients deserve acceptable outcomes with a low incidence of adverse events after one year following contemporary CTO PCI. Strategies solving with proximal ambiguity cap and shortening guide-wire manipulation time are conducive to decreasing TVR.

## Introduction

Percutaneous coronary intervention (PCI) for chronic total occlusion (CTO) patients has become a well-established revascularization procedure in recent years (1, 2). Successful CTO PCI has been shown to effectively relieve symptoms, improve cardiac function, and potentially decrease adverse cardiovascular events (3–5). With advancements in technology and techniques, the procedural success rate now reaches around 95% for experienced operators, making contemporary CTO PCI even more efficient and safer (6).

Previous studies have indicated that left ventricular ejection fraction (LVEF) and CTO lesion length are predictors of outcomes for these specific patients (7, 8). However, few studies have systematically investigated the predictors of mid-term outcomes, particularly the factors related to angiographic characteristics and procedural results. This study aimed to identify and describe the predictive factors influencing clinical outcomes one year after CTO PCI.

## Methods

### Study population

Consecutive patients who underwent CTO PCI at the Department of Cardiology in Xijing Hospital between January 2018 and December 2020 were prospectively included in this study. The Ethics Committee of the First Affiliated Hospital of Air Force Military University approved this study, which was conducted in accordance with the principles of the Declaration of Helsinki, and written informed consents were obtained from all patients. Cases enrolled were limited to those with a proximal CTO vessel diameter of ≥ 2.0 mm. If the patient had undergone multiple CTO PCI procedures, only the first procedure was included for analysis. After discharge, patients entered the follow-up observation period. Exclusion criteria included incomplete data collection, occlusion of bridging vessels, severe infection or malignancy, significant liver or kidney dysfunction which might result in ineligibility for study inclusion, as well as in-hospital mortality and refusal to provide written informed consent.

### Study procedure

The primary indication for CTO PCI was the presence of ischemic symptoms, with or without documented myocardial ischemia. The current guidelines recommend CABG as the first choice for patients with coronary heart disease with a high score of SYNTAX (>32); therefore, surgical consultations were requested for all patients with a SYNTAX score >32 in this study. In some cases, surgeons deemed the patients unsuitable for surgery due to multiple clinical comorbidities or cardiac function <35%. On the other hand, some patients declined surgery due to concerns about the associated surgical risks.

Coronary angiography (CAG) and an in-depth review of the angiogram were routinely conducted before the procedure. The operator took the hybrid algorithm as a guide during planning and performing (9). Other lesions were scheduled to be treated during the index procedure, a subsequent staged procedure in the same admission, or the next hospitalization after one month to achieve complete revascularization, depending on the patients's consent and the operator's discretion. The CTO crossing attempt was about to stop when it reached the termination condition suggested by the CTO crossing algorithm from the Asia Pacific CTO Club (10). All cases received optimal medical treatment according to current guidelines following the index procedure. For a considerable part of the cases with a reference vessel diameter between 2.0 and 2.5 mm, percutaneous transluminal coronary angioplasty (PTCA) with or without a drug-coated balloon (DCB) was performed.

Demographic and clinical data, coronary angiography results, and CTO-PCI outcomes were collected at baseline. The SYNTAX score was calculated using the online scoring system (https://www.syntaxscore.com) to score all coronary artery lesions with a reference diameter ≥ 1.5 mm and lumen diameter stenosis ≥ 50%. The characteristics of CTO lesions involving blunt stump, calcification, bending, occlusion length ≥ 20 mm, and previously failed lesion was evaluated by the variables and definitions of the J-CTO score (11), while proximal cap ambiguity and absence of interventional collaterals were determined by PROGRESS score (12). A poor distal landing zone was defined as the presence of significant coronary artery disease, the existence of a vital bifurcation at the re-entry zone, or a distal vessel diameter < 2 mm. Procedure angiograms were independently evaluated by two experienced interventionists, and clinical follow-up was scheduled at 12 months after CTO PCI via telephone interviews, clinical visits, or re-admission.

### Study definition and endpoint

The primary endpoint was defined as major adverse cardiovascular events (MACE), which included all-cause death and myocardial infarction (MI), and the secondary endpoint was set as target vessel revascularization (TVR) based on the suggestion from the CTO-ARC Consensus Recommendations (13). The diagnosis of MI was based on the Fourth Universal Definition of Myocardial Infarction (14). TVR refers to any repeated percutaneous coronary intervention or surgical bypass of the target vessel.

The guide wire manipulation time (GMT) was defined as the time required to cross the CTO or abort the procedure. The Dissection and re-entry technique (DART) consists of antegrade dissection and re-entry (ADR) and retrograde dissection and re-entry (RDR), which was performed by contemporary dissection and re-entry (DR) operation pattern with the help of dedicated instrument, such as BridgePoint system in ADR, and antegrade balloon dilatation and GuideZilla extended microcatheter in RDR, irrespective of the crossing guidewire intraplaque or extraplaque. Success in this article refers to technical success, which means achieving TIMI grade 3 blood flow with residual stenosis ≤30% of the target CTO lesion. In-hospital complications included periprocedural MI, stroke, emergent repeat TVR with PCI or CABG, life-threatening and major bleeding according to Bleeding Academic Research Consortium criteria, coronary perforation requiring medical or surgical intervention, severe peripheral vascular complications, and acute renal injury.

In view of the predictive factor analysis, several continuous variables were grouped into ordinal variables based on previous research, the latest guidelines, and the specific index procedure. Pro-brain natriuretic peptide (pro-BNP) was categorized into four levels (<221 pg/mL, 221–459 pg/mL, 459–1,006 pg/mL, ≥1,006 pg/mL). LVEF was divided into two groups (<40%, ≥40%) (15). The pre-procedure and residual SYNTAX scores were grouped into two categories based on the 75th percentile (SYNTAX score: <37 points, ≥37 points; residual SYNTAX score: <12 points, ≥12 points). The J-CTO score was classified into two groups (<3 points, ≥3 points). For the procedures which were succeeded with the primary strategy, the median GMT was 20 min, and 80 min for those which were succeeded with the rescue strategy. Therefore, the GMT was divided into three groups (<20 min, 20–80 min, ≥80 min).

### Statistical analysis

Normality was assessed using the Shapiro–Wilk statistic. Numerical values were expressed as mean ± standard deviation (SD) or median [interquartile range (IQR)], as appropriate, and compared using the Student's *t*-test, Mann–Whitney *U*-test, or Kruskal–Wallis *H*-test. Categorical variables were expressed as percentages and compared using the Pearson chi-square test and Fisher's exact test. The Cox proportional hazard model was used to analyze the predictive factors of MACE and TVR. For all tests, a bilateral test probability of *P* < 0.05 was considered to represent a statistically significant difference. All statistical analyses were executed using SPSS version 27 (IBM, Armonk, New York).

## Results

### Study population

**Figure 1:**
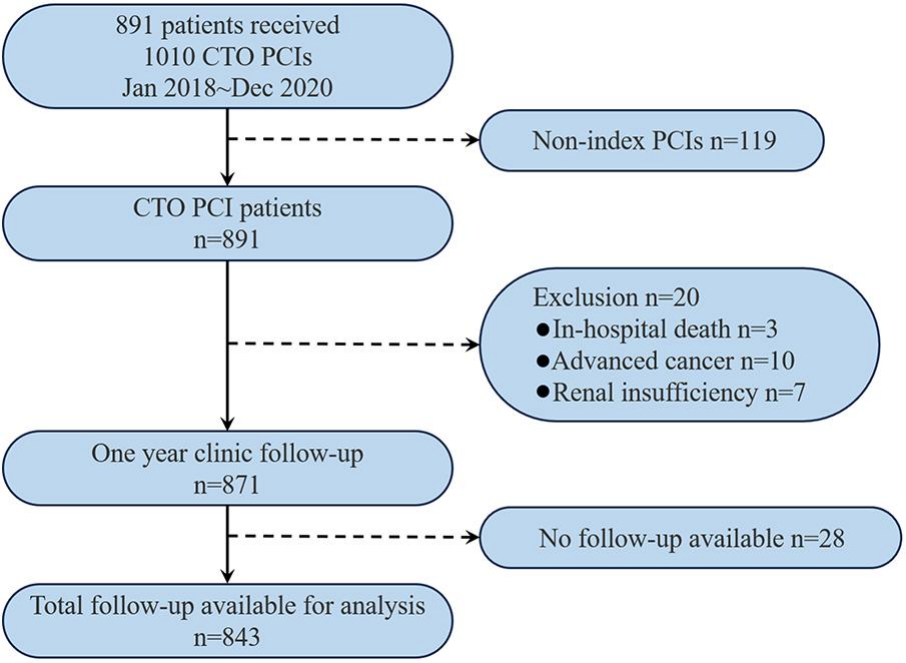
Patient flow chart. CTO, chronic total occlusion; PCI, percutaneous coronary intervention.

A total of 1,010 CTO-PCI procedures were performed in 891 patients from January 2018 to December 2020, of which 119 procedures were excluded as non-index procedures. 848 success cases were achieved among the remaining 891 index CTO PCIs (95.2%). Three cases were excluded due to in-hospital death, ten cases due to advanced cancer, and seven cases due to renal insufficiency requiring permanent dialysis treatment. As for the cohort (*n* = 871), no follow-up data were available in 28 patients; 843 patients (96.8%) constituted the analysis set for the study (Figure 1).

### One-year clinical outcome

The incidence of MACE was observed in 29 patients (3.4%), with all-cause death occurring in 22 cases (2.6%) and MI occurring in 7 cases (0.8%). TVR occurred in 39 patients (4.6%).

### Baseline demographic and clinical characteristics

The mean age was 61 ± 11 years and 732 patients were male (86.8%). Patients in the MACE group had a lower frequency of aspirin usage at discharge (72.4% vs. 95.2%, *P* < 0.001) than those in the no-MACE group. However, they had a higher pro-BNP [676 (211, 3, 017) vs. 319 (117, 960), *P* = 0.018], and a greater prevalence of LVEF < 40% (37.9% vs. 15.5%, *P* = 0.003). In the TVR group, patients had a significantly higher proportion of previous PCI (74.4% vs. 55.8%, *P* = 0.023), a lower rate of LVEF < 40% (2.6% vs. 16.9%, *P* = 0.018), and were less likely to use clopidogrel at discharge (12.8% vs. 28.4%, *P* = 0.034) (Table 1).

**Table 1:** Baseline demographic and clinical characteristics.

Variables	Overall	Primary endpoint			Secondary endpoint		
		non-MACE (*n* = 776)	MACE (*n* = 27)	*P* Value	non-TVR (*n* = 768)	TVR (*n* = 35)	*P* Value
Age, yrs	61 ± 11	61 ± 11	63 ± 11	0.211	61 ± 11	60 ± 12	0.459
Male	732 (86.8)	707 (86.9)	25 (86.2)	0.785	699 (86.9)	33 (84.6)	0.675
BMI, kg/m^2^	25.2 ± 3.2	25.2 ± 3.2	24.0 ± 3.6	0.038	25.2 ± 3.3	24.5 ± 2.8	0.182
Current smoking	309 (36.7)	301 (37.0)	8 (27.6)	0.302	297 (36.9)	12 (30.8)	0.435
Hypertension	503 (59.7)	485 (59.6)	18 (62.1)	0.789	477 (59.3)	26 (66.7)	0.362
Diabetes	286 (33.9)	278 (34.2)	8 (27.6)	0.463	274 (34.1)	12 (30.8)	0.670
Dyslipidemia	506 (60.0)	488 (60.0)	18 (62.1)	0.819	483 (60.1)	23 (59.0)	0.891
Previous MI	338 (40.1)	325 (39.9)	13 (44.8)	0.597	318 (39.6)	20 (51.3)	0.144
Heart failure	83 (9.8)	79 (9.7)	4 (13.8)	0.519	82 (10.2)	1 (2.6%)	0.166
Chronic Kidney Insufficiency	27 (3.2)	27 (3.3)	0 (0.0)	1.000	26 (3.2)	1 (2.6)	1.000
Peripheral vascular disease	25 (3.0)	24 (2.9)	1 (3.4)	0.589	24 (3.0)	1 (2.6)	1.000
Previous PCI	478 (56.7)	466 (57.2)	12 (41.4)	0.090	449 (55.8)	29 (74.4)	0.023
Previous CABG	32 (3.8)	31 (3.8)	1 (3.4)	1.000	29 (3.6)	3 (7.7)	0.180
Creatinine, μmol/L	83 (71, 99)	84 (71, 99)	83 (71, 104)	0.989	84 (71, 100)	79 (70, 89)	0.127
LDL-C, mmol/L	1.7 (1.4, 2.3)	1.7 (1.4, 2.3)	1.8 (1.3, 2.3)	0.824	1.7 (1.4, 2.3)	1.6 (1.3, 2.2)	0.150
pro-BNP, pg/mL	326 (120, 1,012)	319 (117, 960)	676 (211, 3,017)	0.018	336 (123, 1,024)	203 (84, 793)	0.104
pro-BNP grades				0.080			0.249
<221 pg/mL	334 (39.6)	326 (40.0)	8 (27.6)		313 (38.9)	21 (53.8)	
221–459 pg/mL	147 (17.4)	142 (17.4)	5 (17.2)		143 (17.8)	4 (10.3)	
459–1,006 pg/mL	151 (17.9)	148 (18.2)	3 (10.3)		144 (17.9)	7 (17.9)	
≥1,006 pg/mL	211 (25.0)	198 (24.3)	13 (44.8)		204 (25.4)	7 (17.9)	
LVEF, %	53 (44, 57)	54 (45, 57)	50 (36, 55)	0.031	53 (44, 57)	53 (46, 58)	0.324
LVEF < 40%	137 (16.3)	126 (15.5)	11 (37.9)	0.003	136 (16.9)	1 (2.6)	0.018
Medication at discharge							
Aspirin	796 (94.4)	775 (95.2)	21 (72.4)	<0.001	760 (94.5)	36 (92.3%)	0.474
Clopidogrel	233 (27.6)	226 (27.8)	7 (24.1)	0.668	228 (28.4)	5 (12.8)	0.034
Ticagrelor	607 (72.0)	586 (72.0)	21 (72.4)	0.960	574 (71.4)	33 (84.6)	0.072
ACEI/ARB	692 (82.1)	667 (81.9)	25 (86.2)	0.556	663 (82.5)	29 (74.4)	0.197
*β*-blockers	767 (91.0)	739 (90.8)	28 (96.6)	0.506	733 (91.2)	34 (87.2)	0.386
Statin	831 (98.6)	802 (98.5)	29 (100.0)	1.000	793 (98.6)	38 (97.4)	0.436

Values are mean ± SD, median (interquartile range), or *n* (%).

ACEI/ARB, angiotensin-converting enzyme inhibitors/angiotensin II receptor blockers; BNP, brain natriuretic peptide; CABG, coronary artery bypass grafting; LDL-C, low-density lipoprotein cholesterol; LVEF, left ventricular ejection fraction; MI, myocardial infarction; PCI, percutaneous coronary intervention.

### Baseline angiographic characteristics

Nearly half of the patients (44.6%) presented with triple vessel disease, one-fifth of whom (18.3%) had left main trunk lesions. All patients got a high median SYNTAX score [29 (21, 37) points] and a high J-CTO score [3(2,3)] in target lesions. In the MACE group, the SYNTAX score was significantly higher [35(23, 48) vs. 29(21, 37), *P* = 0.023], along with a greater proportion of left main lesions (34.5% vs. 17.7%, *P* = 0.023) and target lesion calcification (58.6% vs. 37.5%, *P* = 0.021), compared to the non-MACE group. In the TVR group, patients had a higher rate of proximal ambiguity cap (53.8% vs. 32.8%, *P* = 0.007) than in the non-TVR group. The proportion of LAD CTO was lower in the non-TVR group (20.5% vs. 38.4%, *P* = 0.024) (Table 2).

**Table 2:** Baseline Angiographic Characteristics.

Variables	Overall	Primary endpoint			Secondary endpoint		
		non-MACE (*n* = 776)	MACE (*n* = 27)	*P* Value	non-TVR (*n* = 768)	TVR (*n* = 35)	*P* Value
Number of diseased vessels							
Single VD	70 (8.3)	69 (8.5)	1 (3.4)	0.503	67 (8.3)	3 (7.7)	1.000
Double VD	243 (28.8)	236 (29.0)	7 (24.1)	0.571	230 (28.6)	13 (33.3)	0.525
Triple VD	376 (44.6)	365 (44.8)	11 (37.9)	0.462	360 (44.8)	16 (41.0)	0.645
LMCA	154 (18.3)	144 (17.7)	10 (34.5)	0.021	147 (18.3)	7 (17.9)	0.958
SYNTAX score SYNTAXscore(Q_L_,Q_U_)]	29 (21, 37)	29 (21, 37)	35 (23, 48)	0.023	29 (21, 38)	26 (17, 32)	0.051
SYNTAX score ≥37	213 (25.3)	200 (24.6)	13 (44.8)	0.014	208 (25.9)	5 (12.8)	0.067
Number of CTO vessels	1 (1, 1)	1 (1, 1)	1 (1, 2)	0.057	1 (1, 1)	1 (1, 1)	0.770
CTO Target vessel							
LMCA	6 (0.7)	6 (0.7)	0 (0.0)	1.000	6 (0.7)	0 (0.0)	1.000
LAD	317 (37.6)	308 (37.8)	9 (31.0)	0.457	309 (38.4)	8 (20.5)	0.024
CX	78 (9.3)	76 (9.3)	2 (6.9)	1.000	73 (9.1)	5 (12.8)	0.396
RCA	442 (52.4)	424 (52.1)	18 (62.1)	0.290	416 (51.7)	26 (66.7)	0.068
In-stent occlusion	68 (8.1)	67 (8.2)	1 (3.4)	0.723	63 (7.8)	5 (12.8)	0.234
Ostial lesion	87 (10.3)	82 (10.1)	5 (17.2)	0.211	82 (10.2)	5 (12.8)	0.588
Blunt stump	590 (70.0)	568 (69.8)	22 (75.9)	0.482	559 (69.5)	31 (79.5)	0.185
Proximal ambiguity cap	285 (33.8)	277 (34.0)	8 (27.6)	0.471	264 (32.8)	21 (53.8)	0.007
Calcification	322 (38.2)	305 (37.5)	17 (58.6)	0.021	308 (38.3)	14 (35.9)	0.762
Tortuosity >45°	611 (72.5)	591 (72.6)	20 (69.0)	0.666	580 (72.1)	31 (79.5)	0.316
reference vessel diameter, mm	3.00 (2.75, 3.50)	3.00 (2.75, 3.25)	3.00 (2.75, 3.50)	0.894	3.00 (2.75, 3.25)	3.00 (2.50, 3.00)	0.164
Lesion length, mm	25 (18, 33)	25 (18, 33)	23 (15, 37)	0.584	25 (18, 33)	30 (18, 38)	0.161
Lesion length ≥20 mm	581 (68.9)	563 (69.2)	18 (62.1)	0.417	553 (68.8)	28 (71.8)	0.691
Lack of interventional collaterals	207 (24.6)	196 (24.1)	11 (37.9)	0.089	194 (24.1)	13 (33.3)	0.192
Diseased distal landing zone	362 (42.9)	353 (43.4)	9 (31.0)	0.187	340 (42.3)	22 (56.4)	0.082
Reattempt	129 (15.3)	125 (15.4)	4 (13.8)	1.000	122 (15.2)	7 (17.9)	0.638
J-CTO score	3 (2, 3)	3 (2, 3)	3 (2, 3)	0.867	3 (2, 3)	3 (2, 4)	0.263
J-CTO score ≥3	500 (59.3)	484 (59.5)	16 (55.2)	0.644	474 (59.0)	26 (66.7)	0.338

Values are mean ± SD, median (interquartile range), or *n* (%).

CTO, Chronic Total Occlusion; CX, Circumflex artery; J-CTO, Japanese Multicenter CTO Registry; LAD, Left Anterior Descending Artery; LMCA, Left Main Coronary Artery; RCA, Right Coronary Artery; SYNTAX, Synergy Between Percutaneous Coronary Intervention with Taxus and Cardiac Surgery; VD, Vessel Disease.

### Procedure characteristics and outcomes

Of the 1,010 CTO PCIs, 950 technique success (94.1%) were achieved, with 848 (95.2%) in the index PCI (*n* = 891) and 102 (85.7%) in the non-index PCI (*n* = 119), which took a higher portion of LCX-CTO as the target lesion (46.2% vs. 9.8%, *P* < 0.001) and a lower inch in the diameter of target vessel [2.75(2.50, 3.00) mm vs. 3.00(2.75, 3.25) mm, *P* < 0.001] than the former.

For the index procedure patients (*n* = 891), 3 cases (0.3%) died respectively from stent thrombosis, cardiac tamponade, and acute myocardial infarction as the main cause of admission. The non-fatal in-hospital complication includes 6 periprocedural myocardial infarctions (0.8%), 2 emergency TVRs (0.2%) (one of whom was referred for CABG), 11 major bleeding (1.2%) (hemopericardium, approach hematoma, and gastrointestinal bleeding), 10 coronary perforations (1.1%) (necessitating the use of pericardiocentesis, covered stents, coils, and thrombin), 18 peripheral vascular complications (2.0%) (mainly resulted from an access-related vascular injury like pseudoaneurysm, arteriovenous fistula, dissection, perforation, pseudoaneurysm, and hematoma), and 2 renal impairments (0.2%).

As in the analysis set (*n* = 843), more than one-half of the procedures (*n* = 549, 65.1%) succeeded with the AW strategy, 215 of which (25.5%) with Retrograde, and the remaining 79 ones (9.4%) with ADR. In the MACE group, the residual SYNTAX score [10(5, 23) vs. 7(3, 12), *P* = 0.012] and the occurrence of hospital complications (13.8% vs. 4.5%, *P* = 0.047) were higher than those in the non-MACE group (Table 3). In this study, there was a significant decrease in the SYNTAX score by 20(12, 27) points in comparing preoperative and postoperative scores. The SYNTAX score in the technique success group showed no difference with the failed group [29(21, 37) vs. 30(24, 41), *P* = 0.177], while the residual SYNTAX score [7(3, 12) vs. 19(11, 26), *P* < 0.001] is lower. Even in the failed group, the residual SYNTAX score is statistically lower (*P* < 0.001) than the SYNTAX score.

**Table 3:** Procedure Characteristics and Outcomes.

Variables	Overall	Primary endpoint			Secondary endpoint		
		non-MACE (*n* = 776)	MACE (*n* = 27)	*P* Value	non-TVR (*n* = 768)	TVR (*n* = 35)	*P* Value
Successful crossing strategy							
AW	549 (65.1)	528 (64.9)	21 (72.4)	0.402	530 (65.9)	19 (48.7)	0.028
Rretro	215 (25.5)	209 (25.7)	6 (20.7)	0.545	201 (25.0)	14 (35.9)	0.127
ADR	79 (9.4)	77 (9.5)	2 (6.9)	1.000	73 (9.1)	6 (15.4)	0.252
DART	226 (26.8)	218 (26.8)	8 (27.6)	0.923	211 (26.2)	15 (38.5)	0.093
GMT, min	30 (10, 63)	30 (10, 63)	35 (12, 66)	0.826	30 (10, 60)	56 (17, 107)	0.003
GMT grades				0.911			<0.001
<20 min	310 (36.8)	300 (36.9)	10 (34.5)		300 (37.3)	10 (25.6)	
20–80 min	383 (45.4)	370 (45.5)	13 (44.8)		370 (46.0)	13 (33.3)	
≥80 min	150 (17.8)	144 (17.7)	6 (20.7)		134 (16.7)	16 (41.0)	
Technique success	803 (95.3)	776 (95.3)	27 (93.1)	0.643	768 (95.5)	35 (89.7)	0.108
Stent implantation	743 (88.1)	717 (88.1)	26 (89.7)	1.000	714 (88.8)	29 (74.4)	0.018
Number of stents	2 (2, 3)	2 (2, 3)	3 (2, 3)	0.431	2 (2, 3)	2 (0, 3)	0.249
Total stent length, mm	72 (46, 99)	72 (47, 99)	77 (46, 105)	0.452	72 (48, 99)	68 (0, 104)	0.478
Contrast volume, mL	350 (270, 430)	350 (270, 430)	320 (235, 445)	0.616	350 (270, 430)	360 (310, 500)	0.062
Residual SYNTAX score	7 (3, 13)	7 (3, 12)	10 (5, 23)	0.012	7 (3, 13)	6 (2, 10)	0.249
Residual SYNTAX score ≥12	245 (29.1)	231 (28.4)	14 (48.3)	0.020	238 (29.6)	7 (17.9)	0.118
Mechanical Circulatory Support	42 (5.0)	38 (4.7)	4 (13.8)	0.051	41 (5.1)	1 (2.6)	0.716
In-hospital complications	41 (4.9)	37 (4.5)	4 (13.8)	0.047	40 (5.0)	1 (2.6)	1.000

Values are mean ± SD, median (interquartile range), or *n* (%).

AW, antegrade wiring; ADR, antegrade dissection and re-entry; DART, dissection and re-entry techniques; GMT, guidewire manipulation time; Retro, retrograde; SYNTAX, synergy between percutaneous coronary intervention with taxus and cardiac surgery.

Compared with the non-TVR group, the TVR group had lower rates of AW strategy success (48.7% vs. 65.9%, *P* = 0.028) and stent implantation (74.4% vs. 88.8%, *P* = 0.018), while longer GMT [56 (17, 107) vs. 30 (10, 60), *P* = 0.003]. Technique success between the MACE and non-MACE group showed no significant difference, but is numerically lower in the TVR group than in the non-TVR group (Table 3).

### Analysis of predictive factors for one-year MACE

The univariate analyses suggested that BMI, LVEF, aspirin usage, left main lesions, SYNTAX score, number of CTOs, calcification, residual SYNTAX score, MCS, and in-hospital complications show correlation with MACE (Table 4).

**Table 4:** Univariate and multivariate analyses of MACE.

Variables	Univariate			Multivariate		
	HR	95% CI	*P* Value	HR	95% CI	*P* Value
BMI	0.880	0.782–0.990	0.033			
LVEF < 40%	3.220	1.521–6.817	0.002	2.889	1.358–6.146	0.006
Aspirin at discharge	0.144	0.064–0.326	<0.001	0.155	0.069–0.352	<0.001
LMCA	2.404	1.118–5.17	0.025			
SYNTAX score	1.051	1.018–1.085	0.002			
Number of CTO vessels	1.903	1.111–3.259	0.019			
Calcification	2.301	1.099–4.818	0.027			
Lack of interventional collaterals	1.956	0.924–4.142	0.080	2.009	0.946–4.265	0.069
Technique success	0.622	0.148–2.616	0.517			
Residual SYNTAX score ≥12	2.322	1.121–4.811	0.023	2.279	1.099–4.724	0.027
MCS	3.165	1.101–9.093	0.032			
In-hospital complications	3.215	1.119–9.239	0.030			

BMI, body mass index; CTO, chronic total occlusion; LMCA, left main coronary artery; LVEF, left ventricular ejection fraction; SYNTAX, synergy between percutaneous coronary intervention with taxus and cardiac surgery; MCS, mechanical circulatory support.

**Figure 2:**
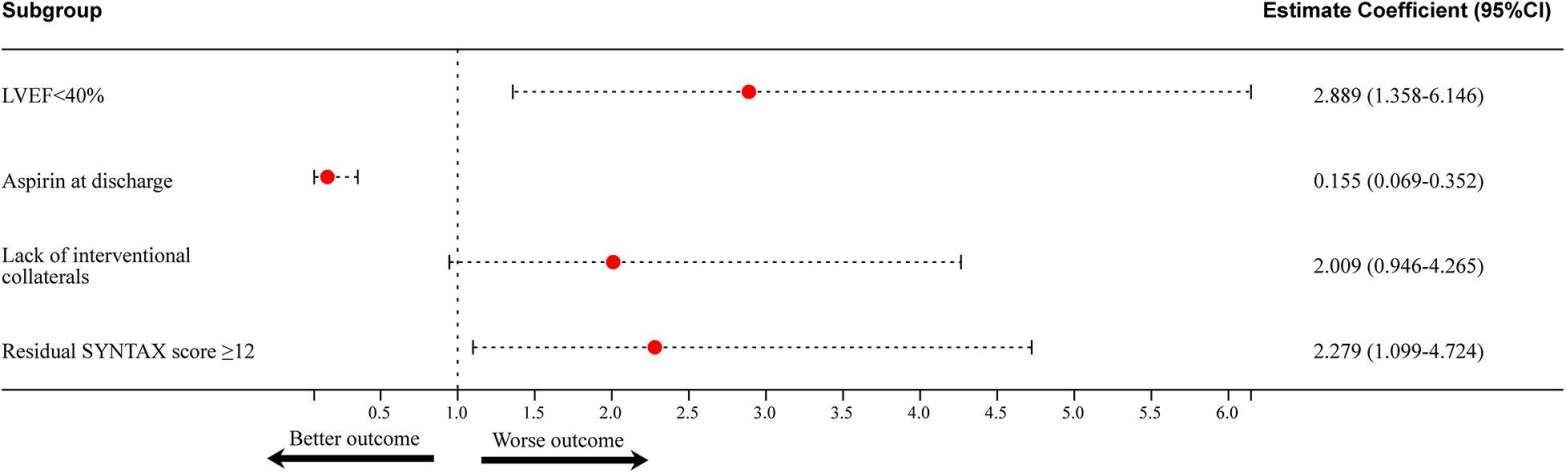
Predictors of one-year MACE. LVEF < 40% and residual Syntax score ≥12 were identified as independent risk factors, while PCI history and aspirin at discharge were found to be protective factors. LVEF, left ventricular ejection fraction; SYNTAX, synergy between percutaneous coronary intervention with taxus and cardiac surgery.

**Figure 3:**
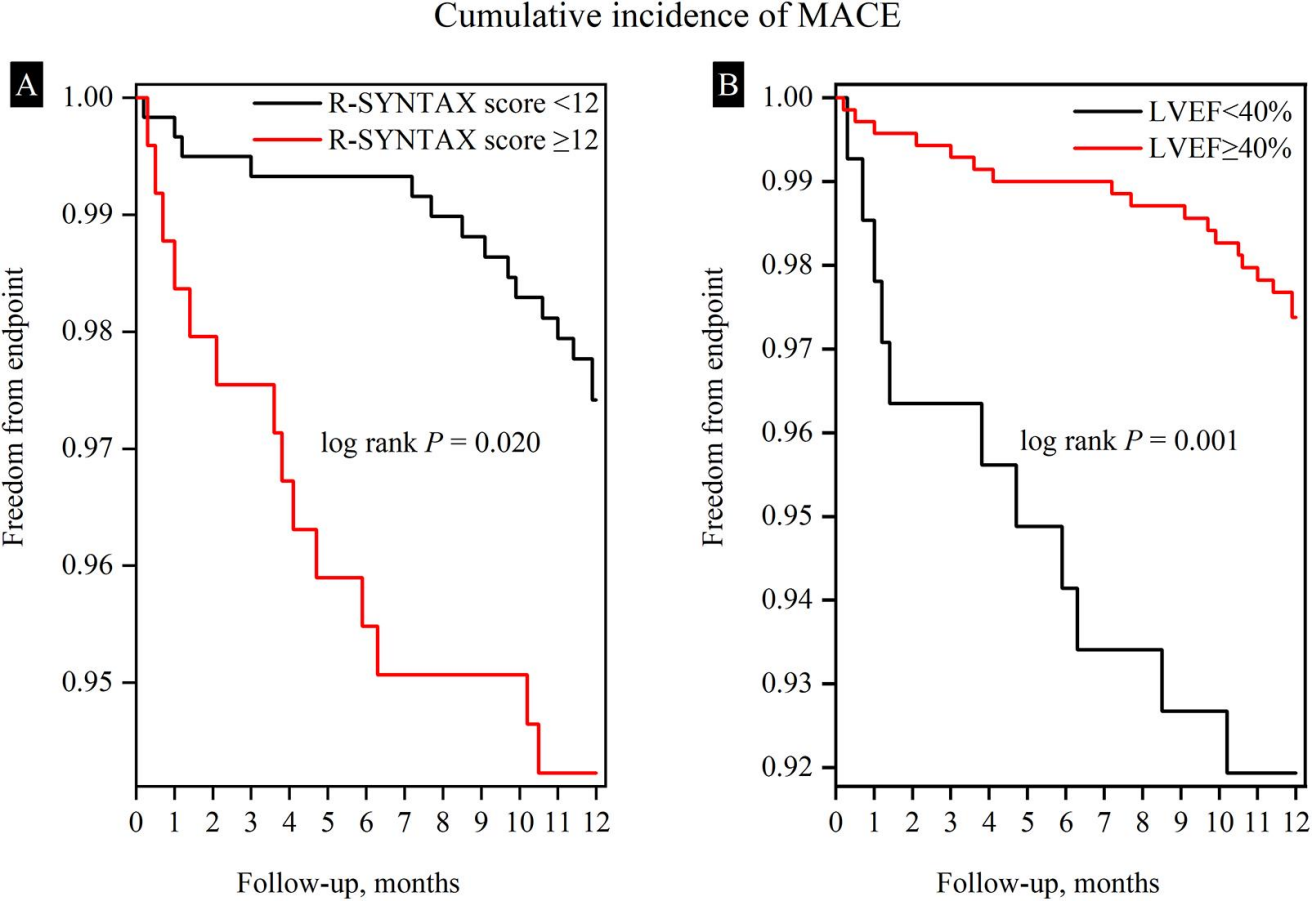
Survival curves for freedom from the cumulative incidence of MACE according to R-SYNTAX score (<12 vs ≥12) and LVEF (<40% vs ≥40%). **(A)** Within one year after CTO PCI, the MACE-free survival rate gradually declined over time in both groups, with a more pronounced decrease observed in the group with an R-SYNTAX score ≥12. The disparity between the two groups demonstrated statistical significance (*P* < 0.05). **(B)** A consistent result according to different degrees of LVEF, the LVEF <40% group showed a more pronounced decrease in the MACE-free survival rate. R-SYNTAX, residual SYNTAX score; LVEF, left ventricular ejection fraction; MACE, major adverse cardiovascular events.

Multivariate analysis showed that LVEF, aspirin usage, and residual SYNTAX score were correlated with MACE. Among these variables, LVEF < 40% (HR = 2.889, 95% CI:1.358–6.146, *P* = 0.006) and residual SYNTAX score ≥12 (HR = 2.279, 95% CI: 1.099–4.724, *P* = 0.027) were identified as independent risk factors, while aspirin usage (HR = 0.155, 95% CI: 0.069–0.352, *P* < 0.001) was found to be a protective factor (Table 4, Figure 2). The Kaplan–Meier plot showed a gradual decline of the MACE-free survival rate over time in both groups after 1-year post-PCI, but a more pronounced decrease was observed in the LVEF < 40% group. The disparity between the two groups demonstrated statistical significance (*P* = 0.001) (Figure 3B). Consequently, an LVEF ≥ 40% conferred a protective effect on patients with CTO compared to those with an LVEF < 40%. Consistently, residual SYNTAX score ≥12 brought more risk to the patient within a year after discharge (*P* = 0.020) (Figure 3A).

### Analysis of predictive factors of one-year TVR

**Figure 4:**
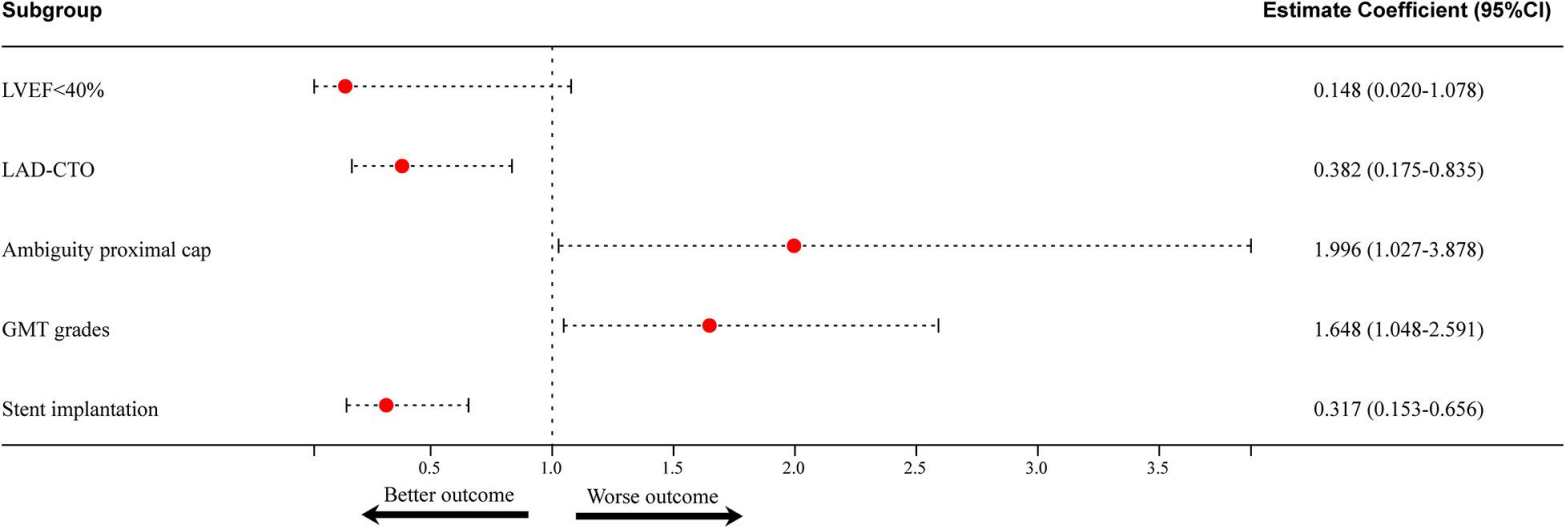
Predictors of one-year TVR. Proximal ambiguity cap and GMT were detected as independent risk factors, while LAD CTO and stent implantation were protective factors. LVEF, left ventricular ejection fraction; LAD, left: left anterior descending (coronary artery); CTO, chronic total occlusion; GMT, guidewire manipulation time.

**Figure 5:**
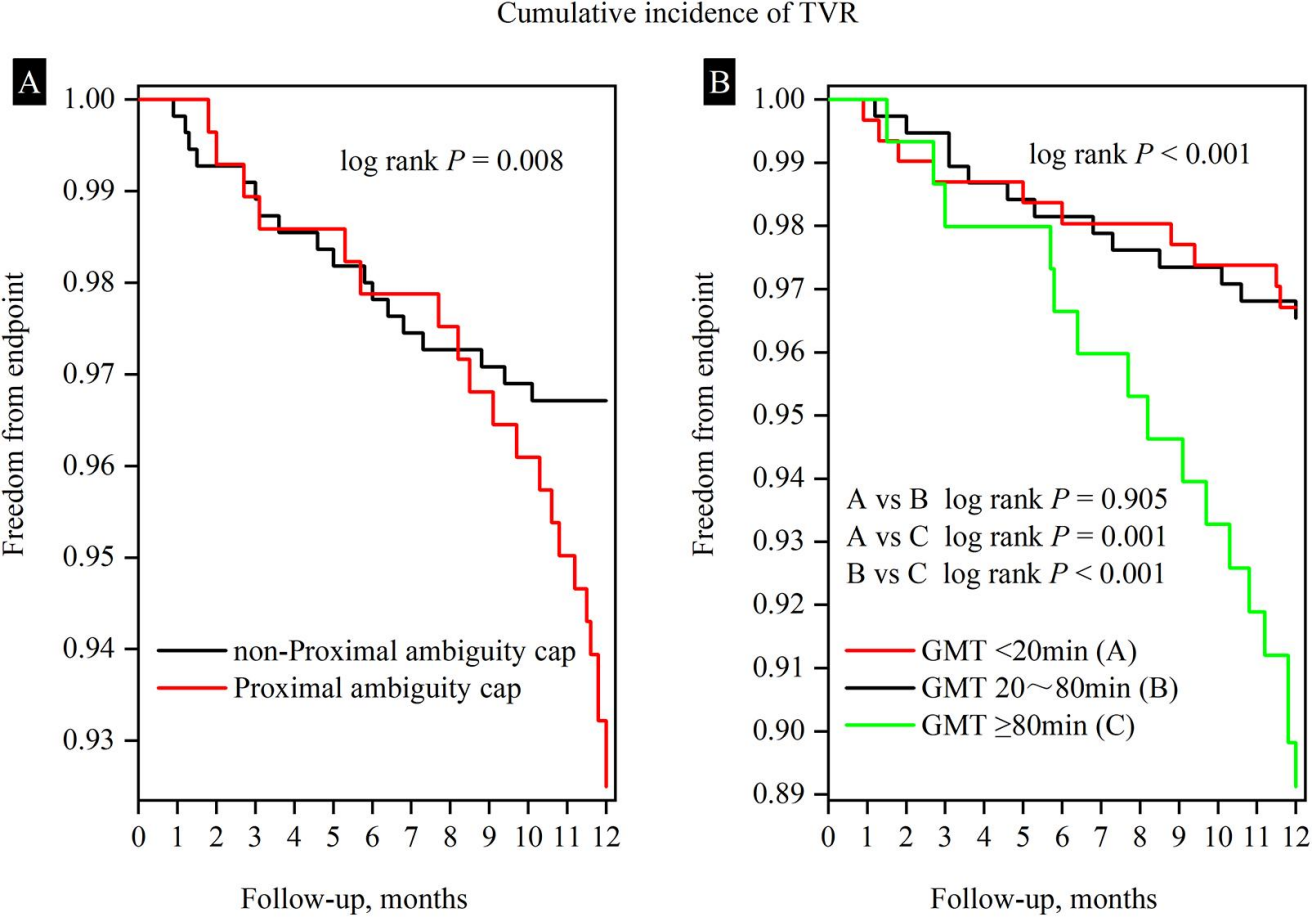
Survival curves for freedom from the cumulative incidence of TVR according to proximal cap (ambiguity vs. non-ambiguity) and GMT (<20 min vs. 20–80 min vs. ≥80 min). **(A)** The 1-year TVR-free survival rate in the non-proximal ambiguity group was higher than that in the proximal ambiguity group. **(B)** Three groups according to GMT exhibit notable variations in their influence on TVR, with the GMT ≥ 80 min group demonstrating the most substantial decline in one-year TVR-free survival rate. Specifically, compared to both the <20 min and 20–80 min groups, the ≥80 min group had a lower rate (*P* < 0.001); however, no statistically significant difference between the former two groups was detected. TVR, target vessel revascularization; GMT, guidewire manipulation time.

Similarly, univariate analysis for TVR suggests that previous PCI, clopidogrel usage, LAD CTO, proximal cap ambiguity, AW crossing, GMT, and DES implantation are significant variables (Table 5). Multivariate analysis showed that proximal cap ambiguity(HR = 1.996, 95% CI:1.027–3.878, *P* = 0.042) and GMT (HR = 1.648, 95% CI:1.048–2.591, *P* = 0.030) were independent risk factors, while LAD CTO (HR = 0.382, 95% CI:0.175–0.835, *P* = 0.016) and stent implantation (HR = 0.317, 95% CI: 0.153–0.656, *P* = 0.002) were protective factors (Table 5, Figure 4). The Kaplan–Meier plot demonstrated that the 1-year TVR-free survival rate in the non-proximal ambiguity group was higher than that in the proximal ambiguity group (*P* = 0.008) (Figure 5A). The impact of GMT on TVR was further confirmed and the results indicated no statistical difference between the<20 min group and the 20–80 min group, while significant differences were observed between the ≥80 min group and both the <20 min group and the 20–80 min group (*P* < 0.001) (Figure 5B).

**Table 5:** Univariate and multivariate analyses of TVR.

Variables	Univariate			Multivariate		
	HR	95% CI	*P* Value	HR	95% CI	*P* Value
Previous PCI	2.222	1.083–4.56	0.029			
LVEF < 40%	0.139	0.019–1.015	0.052	0.148	0.020–1.078	0.059
Clopidogrel at discharge	0.379	0.148–0.969	0.043			
SYNTAX score	0.972	0.943–1.002	0.065			
LAD-CTO	0.416	0.191–0.906	0.027	0.382	0.175–0.835	0.016
Ambiguity proximal cap	2.276	1.213–4.272	0.010	1.996	1.027–3.878	0.042
Diseased distal landing zone	1.713	0.91–3.225	0.096			
AW for crossing strategy	0.512	0.273–0.959	0.037			
DART	1.695	0.889–3.231	0.109			
GMT grades	1.948	1.257–3.017	0.003	1.648	1.048–2.591	0.030
Technique success	0.399	0.142–1.122	0.081			
Stent implantation	0.365	0.178–0.749	0.006	0.317	0.153–0.656	0.002

AW, antegrade wiring; CTO, chronic total occlusion; DART, dissection and re-entry techniques; GMT, guidewire manipulation time; LAD, left anterior descending artery; LVEF, left ventricular ejection fraction; PCI, percutaneous coronary intervention.

## Discussion

In recent years, several studies have reported a significantly high success rate of CTO PCI, making it an increasingly accepted treatment for patients with CTO (6, 16). However, limited evidence was found to identify prognostic factors, particularly those related to lesion characteristics and procedure outcomes, that would impact the one-year outcome of patients treated with contemporary CTO PCI. Therefore, we analyzed to identify predictive factors that may influence the one-year outcomes. In this study, the patients had a high proportion of co-morbidities, multivessel disease, and complex CTO lesions. Most procedures succeeded with the AW strategy, while ADR was utilized in only a minority of cases. Overall, the procedures were performed with a shorter guidewire manipulation time compared to the report from the Japanese CTO-PCI expert registry (17) and with a lower rate of stent implantation compared to the data from the RECHARGE registry (18), which is mainly due to smaller reference diameters. The incidence of MACE and TVR in the follow-up results was parallelly low, similar to previously published data (19, 20).

According to the multivariable analysis, strategy and DART performed during the procedure were not associated with the endpoint of MACE. Instead, LVEF, aspirin usage at discharge, and residual SYNTAX score played a significant role. This study found that all-cause mortality and MACEs were higher in patients with LVEF < 40% compared to those with LVEF ≥ 40% after CTO PCI. What needs to be remembered is that reduced LV function was associated with a 3-fold increased risk of mortality in patients with CTO lesions undergoing PCI, and patients with reduced LV function also could deserve clear benefits of all-cause mortality reduction after successful CTO PCI than those with procedure failure (21). This is important for the relatively young population, especially with LV dysfunction. Furthermore, six patients who were not prescribed aspirin at discharge died. These patients were categorized based on their previous gastrointestinal bleeding (2 cases), age ≥75 years (1 case), severe anemia resulting in massive blood loss during the procedure (1 case), and use of oral anticoagulation (2 cases). Although the cause of death for these patients couldn't be determined, it was an inevitable problem that cases in the MACE group had a higher proportion of not taking aspirin upon discharge. Therefore, it is important to balance antiplatelet therapy with the risk of bleeding for a special population after CTO PCI. CTO lesions are accompanied by multivessel lesions. Our study results showed that incomplete revascularization was significantly associated with a higher incidence of MACEs, aligning with previous findings (22, 23). Therefore, the lower the residual SYNTAX score in CTO PCI, the greater the reduction in MACE. In our study, the residual SYNTAX score was relatively low because of non-index coronary lesions being treated staged on schedule. Even in the index CTO PCI failed group, there was a significant decrease in comparing the preoperative and postoperative scores. This is the main reason for them to achieve nearly equal outcomes in following up.

Multivariable analysis for TVR revealed that the occurrence of TVR was correlated with LAD-CTO, proximal cap ambiguity, GMT, and implanted stents. Patients with a LAD-CTO are prone to having a lower rate of TVR due to less anatomical tortuosity and less frequent DART employed. But, the impact of DART on TVR remains controversial (24). Several reports have suggested that the use of DART is correlated with an increase in TVR. While in this study, the frequency of TVR in the DART group was not significantly higher than that in the wire-based group, but there was a numerical increase.

Besides the above predictive factors explored by current studies, we found that the proximal ambiguity cap of CTO lesion and guide-wire manipulation time significantly impact the TVR after CTO PCI, which has rarely been detected previously. Proximal ambiguity cap is universally recognized as an important adverse characteristic of CTO lesion, for which the retrograde technique is recommended as the primary strategy, given the presence of interventional collateral (9, 12). For interventional collateral unusable, ADR may be the only option (25). In this study, the proximal ambiguity cap significantly increased TVR. Procedures with proximal ambiguity cap were associated with longer lesion lengths, more frequent use of DART, and more guide-wire and device manipulation. To some extent, longer subintimal tracking and more devices applied in the CTO segment tend to result in a wider subintimal space, which implies more loss of endothelial cells and desmoplasia, leading to restenosis (26, 27). The GMT practically indicates the complexity of CTO lesions. The long GMT means a high J-CTO score lesion, and more complex techniques and invasive devices are needed. All these characteristics potentially contribute to a wide subintimal space, injuring the vascular structure and connective tissue hyperplasia (28). During the procedure and following up, more complications may occur (28).

In conclusion, we conducted a systematic predictive factors analysis for patients treated with CTO PCI driven by the development of devices and technologies in past decades, involving clinical baselines, angiographic characteristics, and procedural outcomes. Patients enrolled had a severe chronic heart disease and a heavy lesion burden in the coronary arteries. The operator performed procedures with hybrid strategies and advanced devices, with a high success rate and low in-hospital and follow-up outcomes. The results verified the factors explored by current studies, and detected angiographic characteristics and procedural outcomes impacting the outcome as well, like proximal ambiguity cap and guide-wire manipulation time, which is interesting and rarely researched. While these findings require confirmation by a large-scale, multicenter randomized controlled trial with long-term follow-up.

### Study limitations

This is a prospective single-center observational study, which is limited by its inherent susceptibility to selection bias. Each CTO PCI procedure was performed by an experienced interventional cardiologist; caution should be exercised when applying these results to clinical practice. Also, there is no committee for adverse event adjudication. However, considering the purpose of this study, coronary angiographic characteristics and procedure outcomes from a single operator offer consistent data scales, reduce operator bias, and enhance the stability of the results.

## Conclusions

Patients deserve acceptable outcomes with a low incidence of adverse events after one year following contemporary CTO PCI. LVEF <40% and residual SYNTAX score ≥ 12 were risk factors for MACE, and proximal ambiguity cap and guide-wire manipulation time were risk factors for TVR.

## Data Availability

The original contributions presented in the study are included in the article/Supplementary Material, further inquiries can be directed to the corresponding author/s.
